# Flow and heat transfer of Poly Dispersed SiO_2_ Nanoparticles in Aqueous Glycerol in a Horizontal pipe: Application of ensemble and evolutionary machine learning for model-prediction

**DOI:** 10.1371/journal.pone.0323347

**Published:** 2025-06-03

**Authors:** K. V. Sharma, M.T. Naik, Praveen Kumar Kanti, D.M. Reddy Prasad, Prabhu Paramasivam, Abinet Gosaye Ayanie

**Affiliations:** 1 Centre for Energy Studies, Jawaharlal Nehru Technological University, Hyderabad, Telangana, India; 2 University Center for Research & Development (UCRD), Chandigarh University, Mohali, Punjab, India; 3 Petroleum and Chemical Engineering Programme Area, Faculty of Engineering, Universiti Teknologi Brunei, Gadong, Brunei Darussalam; 4 Department of Research and Innovation, Saveetha School of Engineering, SIMATS, Chennai, Tamil Nadu, India; 5 Department of Mechanical Engineering, Adama Science and Technology University, Adama, Ethiopia; Koneru Lakshmaiah Education Foundation / Indian and Xidian University, INDIA

## Abstract

Stable nanofluid dispersion with SiO_2_ particles of 15, 50, and 100 nm is generated in a base liquid composed of water and glycerol in a 7:3 ratio and tested for physical characteristics in the temperature range of 20-100^o^C. The nanofluid showed excellent stability for over a month. Experiments are undertaken for the flow of nanoﬂuid in a copper pipe and measured for their heat transfer coefficient and ﬂow behavior. The convection heat transfer coefficient increases with the flow Reynolds number in the transition-turbulent flow regime. The experimental results further reveal that the friction factor enhancement with 0.5% concentration has increased by 6% as compared to the base liquid. It was employed for prognostic model development using XGBoost and multi-gene genetic programming (MGGP) to model and predict the complex and nonlinear data acquired during experiments. Both techniques provided robust predictions, as witnessed by the statistical evaluation. The R^2^ statistics of the XGBoost-based model was 0.9899 throughout the model test, while it was lowered to 0.9455 for the MGGP-based model. However, the change was insignificant. The mean squared value was 8.37 for XGBoost, while it increased in the MGGP model to 45.12. Similarly, the mean absolute error (MAE) value was higher (6.623) in the case of MGGP than in XGBoost at 2.733. The statistical evaluation, Taylor diagrams, and violin plots helped determine that XGBoost was superior to MGGP in the present work.

## 1. Introduction

A ﬂuid medium is used to add or remove heat from a system. The rate and amount of heat transported are established to ensure the system’s operation and reliability [1]. For many decades, heat transfer fluids like water, ethylene glycol (EG), and glycerol have been widely used in many different industries such as power generation, manufacturing of chemicals, transport, and microelectronics for homes and workplaces (e.g., air conditioning, refrigeration, and central heating). A fluid’s performance depends on its physical properties and interaction with the environment for heat transfer [2]. The heat transfer coefficients with conventional ﬂuids require large surface areas and high pumping power. Many authors have developed high-performance heat transfer ﬂuids in recent decades.

Nanofluids are colloids prepared by dispersion of nanoparticles (1–100nm) in a base liquid, which show a substantial increase in thermal conductivity (TC). Research has documented the production of stable nanofluids through a two-step procedure [3,4]. The prospective advantages of accurately engineered nanofluids include (i) higher TC compared to anticipated by present-day macroscopic models, (ii) outstanding stability, and (iii) a minor penalty owing to a rise in pressure drop along with pipe wall abrasion encountered by nanometer-sized particle suspensions [5,6].

Determining the forced convection heat transfer coefficient considers the fluid TC and other properties directly or indirectly through the Nusselt number. Most research is directed toward determining TC and viscosity as they are easier to measure than heat transfer coefficients. However, the crucial indicators of improved heat transfer performance in test nanofluids are the enhancements in effective TC and changes in specific heat (SH), density, and viscosity [7]. The determination of heat transfer coefficients ultimately determines the overall advantage of using these fluids. The authors observed that nanofluid heat transfer coefficients increased above the mere TC impact and vice versa.

Nanofluid heat transfer capabilities may be determined by employing the Mouromtseff number (Mo). The thermophysical properties of the base liquid and nanofluid at various temperatures are considered in its evaluation. Another approach to evaluating a nanofluid’s heat transfer capability is by assessing the Property Enhancement Ratio, PER, which relates viscosity to TC enhancements with temperature. Silica (SiO_2_) is a complex ceramic oxide mainly found in silica sand, sandstone, and quartzite. SiO_2_ was selected due to its outstanding features, such as low density, good resistance to abrasion, electric conductivity, and thermal stability, with TC ranging from 1.1 to 1.4 (W/m-K). Furthermore, despite surfactant reinforcement, SiO_2_ colloids are soluble in various polar solvents. Utilizing silica to enhance the properties of liquids holds possible applications in the field of thermal energy.

At this stage, comprehending the characteristics of SiO_2_ nanofluids, precisely their viscosity, density, TC, and SH, will considerably improve the design of thermal process systems. The heat transfer potential of nanofluids is quantified by SH capacity, which is considered a heat transfer parameter. **Murshed** [8] reported the SH of TiO_2_ (15nm)-EG. It has been found that the SH of nanofluid reduces as the concentration of nanofluid increases. **Cabaliero et al.** [9] reported the relation between temperature and the SH of nanofluid for MgO (35nm), ZnO (40–100nm), and ZrO_2_ (30–60nm)/EG in the range of 30^o^C and 200^o^C. It was observed that the rise in SH of the nanofluid was not affected by temperature. The nanofluids containing ZnO, MgO, and ZrO_2_ with particle loading of 2.5 wt.% showed a mean temperature-based rise of 8.5%.

Higher viscosity usually leads to an ascent in pressure drop and a reduction in Nusselt number (Nu) [10]. Therefore, it is crucial to evaluate the viscosity in convective fluxes. Research has been undertaken to analyze the rheological properties of different nanofluids. However, there are still discrepancies in the findings reported. An illustrative instance involves an everyday circumstance where certain research groups identified nanofluids as Newtonian fluids [11–13], whereas others branded them as non-Newtonian [14,15]. However, **Li et al.** [16] recently related the behavior of the nanoparticles to internal disturbances induced by factors like concentration, rate of shear, and particle agglomeration. **Rudyak et al.** [17] stated that the viscosity of SiO2 (18nm)/EG nanofluid rose as the particle concentration increased. Furthermore, **Tadjarodi and Zabihi** [12] reported comparable findings when studying glycerol-based dispersions of mesoporous silica nanofluid.

The TC of additives is an important characteristic that has garnered much attention. It exhibits a significant impact on cooling applications compared to other transfer qualities [18]. **Through ultrasonication and stirring techniques, Tadjarodi and Zabihi** [12] synthesized a stable nanofluid that included mesoporous SiO_2_ (66nm) and glycerol. Their findings showed that the TC improves by roughly 9.24% when the particle concentration is between 2 and 4 wt.%, and it exhibits a mild dependence upon temperature, with conductivity gains observed between 30 and 50^o^C. **Xie et al.** [19] reported the TC data for various oxide nanofluids utilizing ethylene glycol (EG), including Al_2_O_3_, SiO_2_, TiO_2_, MgO, and ZnO. The average particle size used in the research they conducted was 20nm. The researchers observed improved TC when the 5.0 vol.% loading of nanoparticles was maintained. **Kanti et al.** [20] reported enhanced viscosity and TC of water-based nanofluid at 6.38 and 14% at 30 and 60, respectively, with a fly ash concentration of 0.5% in 61% SiO_2_. The studies indicate that a mixture of EG and Water can have higher TC and lower viscosity than EG.

The density of the nanofluid is an additional characteristic that impacts both the pressure drop and friction coefficient. At a temperature of 298 K, **Pak and Cho** [21] investigated the influence of SiO_2_/water nanofluid density over the nanofluid concentration. The density aspect was explored by **Vajjha et al.** [22] for SiO_2_, TiO_2_, and Al_2_O_3_ nanofluids using a mix of water and ethylene glycol in a ratio of 40:60. Most heat transfer studies have focused on using primary liquids that include water, engine oils, EG, and glycerol. Both EG and glycerol have a significantly greater viscosity when compared to water. Consequently, their combinations with water in various proportions are then used to ascertain the characteristics of the base and nanofluid.

Machine learning (ML) is a promising tool for forecasting nanofluids’ convection heat transfer parameters. These nanofluids, composed of nanoparticles distributed in a base fluid, have increased thermal properties, making them intriguing candidates for improved heat transfer technologies [23]. The modeling process starts with data collection from tests or simulations, which includes critical parameters like nanoparticle concentration, rate of fluid flow, temperature, and TC. After that, feature selection is employed to discover critical heat transfer parameters [24].

ML offers significant advantages over traditional empirical or mechanistic models for predictive purposes. It can capture complex, nonlinear relationships in data, providing more accurate predictions without relying on simplified assumptions. ML models adapt directly from experimental data, making them flexible across different conditions, while traditional models require specific physical equations. Additionally, machine learning is faster, handling large datasets efficiently and offering real-time predictions. Its ability to manage multiple variables at once leads to better generalization across various scenarios. As ML models learn from new data, they continuously improve, making them highly effective in dynamic, evolving applications.

The extensive evaluation of correlations among various parameters is then captured using regression models like neural networks, decision trees, or ensemble approaches like random forests. Deep learning, predominantly neural networks, offers a sophisticated technique for detecting complicated patterns in complex datasets. Investigators can gain insights into the importance of many factors, helping them comprehend the complex physics of heat transfer in SiO_2_ nanofluids. Also, the model’s interpretability provides a thorough understanding of its internal workings. Once the prediction model has been trained, the real-time forecast becomes possible, offering valuable insights for experimental design and process optimization, eventually helping to improve performance and efficiency in SiO_2_ nanofluid convection heat transfer applications [25–27].

Water shows a lower viscosity and more excellent TC when compared with glycerol, and it can form a homogeneous mixture with glycerol. Literature suggests that glycerol can draw in and hold moisture from the surrounding atmosphere, possibly surpassing its weight by over twofold. In a prior study by **Prasad et al.** [28], a mix of aqueous glycerol and SiO_2_ dispersions was used. The mixture had a ratio of 3:7, with the glycerol weighing under half of the water weight. The SiO_2_ dispersions had a mean particle size of 50nm. The investigators conducted experiments to determine the viscosity and TC of the test nanofluid. They then employed a mixing relation to estimate the SH and density of the test nanofluid. There are still discrepancies in the conclusions that were offered. An illustrative example includes a common scenario wherein certain research groups categorized nanofluids as Newtonian fluids.

Experiments have been performed to investigate this phenomenon by measuring the thermophysical characteristics of nanofluids, pressure drop, and heat transfer coefficients. The present study employs SiO_2_ nanoparticles with a mean diameter of 15, 50, and 100 nm, which are disseminated in the 30 GW base liquid. The thermophysical parameters of a nanofluid combination, including TC, SH, viscosity, and density, were determined using experiments for SiO_2_ concentrations of 0.5 and 1.0 vol.%. The influence of particle size on the TC of the nanofluid is thoroughly examined to verify the validity of the combination comprising three different particle sizes.

Most of the work reported in the literature is undertaken with water for the determination of heat transfer coefficients and pressure drop. Very limited research has been undertaken with base liquid aqueous glycerol. Further, earlier works with SiO_2_ have been undertaken with average particles reporting of a single particle size. However, no work has been with undertaken to the best of authors’ knowledge to determine their effect on heat transfer coefficient with particles of different sizes. Hence, it is intended to study the influence of poly dispersed SiO_2_ nanofluid on heat transfer and pressure drop.

The formulation and characterization of stable SiO_2_ nanofluids are carried out, followed by tests to investigate nanofluids’ flow and heat transfer characteristics in a straight horizontal pipe under turbulent conditions within a specific range of Reynolds numbers (Re). The motivations behind this are limited literature on (a) the influence of base liquid 30GW on convective heat transfer of SiO_2_ nanoﬂuids (b) the eﬀect of poly dispersion SiO_2_ nanoparticles in aqueous glycerol on the convective heat transfer performance and friction factor with the properties determined through experiments. Two different ML approaches, i.e., ensemble-based XGBoost and evolutionary approach-based multi-gene genetic programming (MGGP), were employed for model prediction of pressure drop and heat transfer. An extensive comparative analysis uses statistical and pictorial methods like Taylor’s graph.

## 2. Materials and methods

### 2.1 Materials and nanoﬂuids formulation

**Fig 1:**
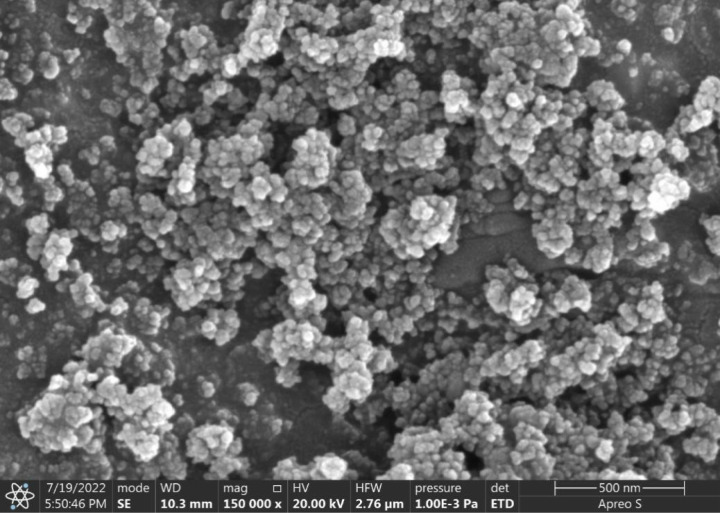
SEM Image of SiO_2_ nanoparticles of average size 15nm.

The nanofluid was produced by combining SiO_2_ nanoparticles with aqueous glycerol in two steps. The initial SiO_2_ nanoparticles were spherical, having mean diameters of 15, 50, and 100nm, made by the Nano Research Laboratory in New Delhi and utilized in their original form. Molychem in Mumbai provided analytical-grade glycerol (C_3_H8O_3_, purity 99.9%). Distilled water was obtained locally from AMS Enterprises in Hyderabad. **Fig 1** shows an SEM picture of the sample.

### 2.2 Preparation of nanofluid

Water and glycerol in a 7:3 ratio mixture by weight (30GW) was prepared as the base fluid having a pH of 4.26. Nanoparticles containing 1.206 and 2.424grams of 15, 50, and 100nm were dispersed individually in 100ml of the base liquid corresponding to 0.5% and 1.0% concentration by volume (SiO_2_ density is 2400 kg/m^3^). Three nanofluids comprising particles spanning 15nm and 6.5 pH, 50nm and 7.19 pH, and 100nm and 8.12 pH correspondingly, stirred together employing a magnetic type stirrer operating at a speed of 750 rpm for two hours. The goal is to generate a uniform blend. Subsequently, high frequency ultrasonication is employed for two hours to achieve a uniform nanofluid with concentrations ranging from 0.5% and 7.25 pH to 1.0% to 7.32 pH, respectively. No sediments of particles were identified during the ocular examination of the samples 30 days after preparation.


$$\phi =\frac{\frac{m_{SiO_{2}}}{\rho_{SiO_{2}}}}{\frac{m_{SiO_{2}}}{\rho_{SiO_{2}}}+V_{30GW}}x100\;$$ (1)

Herein, ϕ denotes the percentage particle volume concentration, V represents the volume of base fluid, and ρ and *m* denote the density and mass of the test nanoparticles, respectively.

### 2.3 Characterization and morphology

FESEM, EDX, FTIR, UV Visual Spectroscopy, and zeta potential for morphology and dispersion stability characterize the samples of 0.5% and 1% concentration. The shape and structure of the nanoparticles with a size of 15nm and coated with SiO_2_ were analyzed using a variable pressure FESEM “Supra 55VP, Carl Zeiss, Germany”. The FESEM image shows the particles to be spherical. Malvern Zeta Size instrument that uses the Dynamic Light Scattering (DLS) approach is utilized to determine the Z-average particle size of 2904nm. The EDX analysis provided a comprehensive sample mapping and reported Si and Oxygen to be 75.17 and 24.83% by weight. The zeta potential was more significant than 30mV with pH between 7 and 8, ensuring nanofluid stability. As the nanofluid contains particles of 15, 50 and 100nm, the average size is estimated to be about 72nm. The UV visible spectroscopy assessed the stability of the colloidal suspension and determined the peak absorptance of the three-particle nanofluid solution to occur at the same wavelength of 265nm.

### 2.4 Properties of SiO_2_ nanoﬂuid

The Anton-Paar instrument (Model: DMA 35 Basic) measured the liquid density of the stabilized nanofluid. Model TPS 500S of Hot Disk, Thermal Constants Analyzer, was utilized to determine the TC, SH capacity, and diffusivity of both test nanofluid and base liquid. This instrument works on the Transient Plane Source (TPS) concept. The DV2T Cup, as well as the Cone (Spindle) Brookfield viscometer, was utilized to determine the magnitude of the viscosity of the nanofluid being tested. The instruments are linked to computer software installed for display and recording. The base liquid and nanofluid properties are reported by Sharma et al. [29].

Recent studies with nanofluids are directed in the low concentration range of < 1.0 vol%, as the particles do not easily agglomerate with the aqueous glycerol base liquid. The result suggests that the presence of nanoparticles in the flow has an even more significant effect on heat transfer compared to what would be anticipated only based on the increase in TC. **Johnson et al.** [30] **and Aoki et al.** [31] have concluded that this is owed to the migration of particles in the fluid stream, aiding heat transfer between the wall surface and the fluid. Evaluation of properties and heat transfer coefficients with SiO_2_ nanofluid dispersed in aqueous glycerol is scarce and undertaken for the first time.

### 2.5 Experimental apparatus

**Fig 2:**
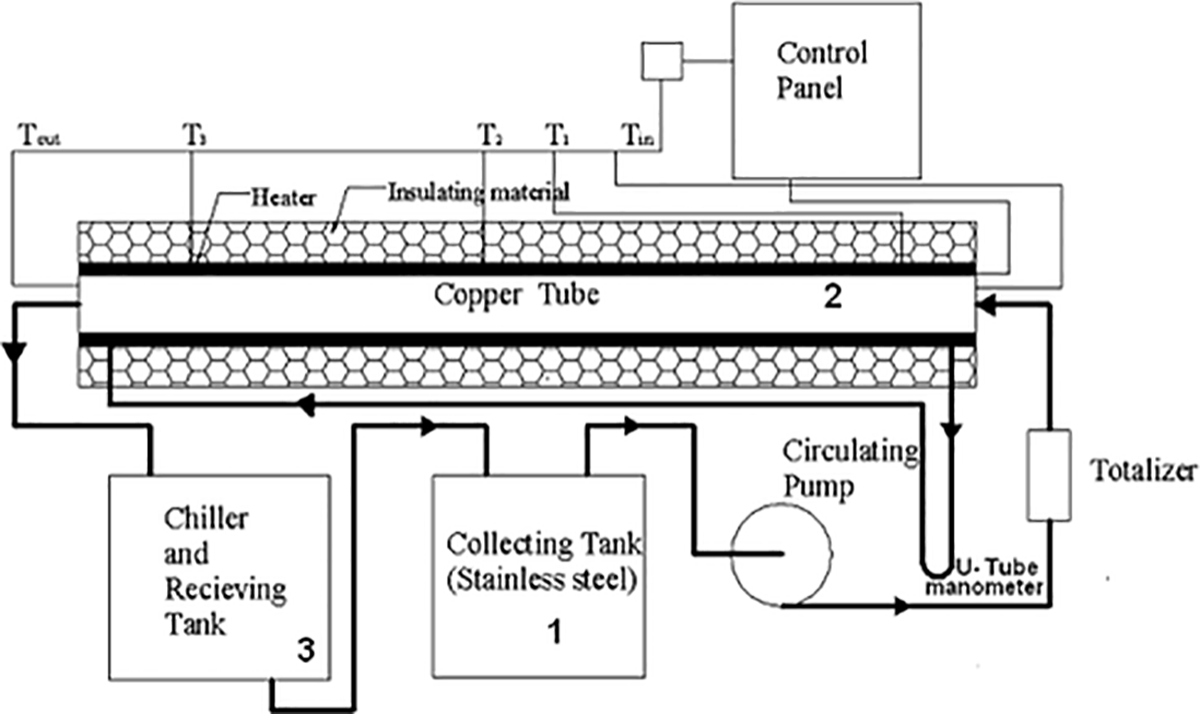
Schematic diagram of the test setup.

The representation diagram of the test setup is presented in **Fig 2**. The experimental design comprises a circulating pump, flow measuring device, chiller, collecting tank, 1.5 m Copper tube test section, flexible heater, control panel, thermocouples, and manometer. The heater encloses the horizontal copper tube of 1.35m (inner diameter = 10 mm and outer diameter = 12 mm), constituting the test section. The length of fluid flow before its entry in the test section is approximately 1.0m, ensuring turbulent flow conditions at the entry of the test section.

A pump with adjustable speed having rated power as 0.5 HP was connected to a stainless-steel tank with a capacity of 0.02 m^3^. The objective was to gather and circulate the working fluid throughout the test area. Two nichrome heaters, each rated at 400W, are wrapped about the outer circle of the test area. There are five K-type thermocouples placed at various points. Three of them are placed on the surface of the tube at distances of 0.40m, 0.65m, and 0.95m from the inlet. The other two thermocouples are set at the inlet and outlet, with a distance between them of 1.35m, allowing for the monitoring of fluid temperatures. The test part and the heaters are enveloped in asbestos rope insulation to limit thermal dissipation to the surrounding environment. The flow rate is chosen before the liquid enters the storage container. The data logger captures the fluid’s temperatures and surface at intervals of 65 seconds to determine the experiment’s steady-state condition.

The heating supply and pump speed can both be individually adjusted employing dimmerstats. The pump speed is adjusted to control the rate of fluid flow. The experimental equipment is used to conduct experiments on distilled water to measure the pressure drop and heat transfer coefficients at different flow rates. Under steady-state conditions, the flow rate and pressure reduce throughout the tube length, and fluid temperature at the intake and output and surface temperatures are measured. The measured heat transfer coefficient and friction factor are determined by the relationships of Darcy’s law and Newton’s law of cooling, respectively. The experimental data acquired with water is cross-validated using established relationships retrieved from the literature to assure the reliability of the test configuration. It enables investigations with the base liquid 30GW and SiO_2_ nanofluid at 0.5% concentration at various flow rates. The coefficient of convective heat transfer, denoted as h in Equation (2), is commonly represented by the Nu:


$$Nu=\frac{hD}{k_{f}}$$ (2)

Herein, the inner dia. of the test tube is denoted with, and *k*_f_ represents TC of test ﬂuid.

### 2.6 Machine learning

Python-based libraries were used to develop pressure drop and heat transfer models using data collected in lab-based experiments. The present study employed an evolutionary approach based on the multi-gene genetic programming (MGGP) machine learning technique and ensemble-based extreme gradient boosting (XGBoost).

#### 2.6.1 Multi gene genetic programming.

MGGP is a modern ML technique based on genetic programming and evolutionary algorithms. It is a subset of a larger class of symbolic regression techniques that seeks to expose mathematical expressions articulating data relationships [32]. The fundamental idea of MGGP is to encode mathematical expressions as chromosomes in a population of alternative solutions. Every response is a potential mathematical model. The algorithm employs genetic operators like crossover and mutation to evolve these solutions iteratively.

The following key steps are involved in the evolutionary process:

- Initialization: A population of random mathematical expressions representing potential models is formed.
- Evaluation: Each model in the population is scored depending on how well it fits the training data. This assessment entails calculating a fitness score representing how well the model explains the observed results.
- Models are chosen for reproduction depending on their fitness scores. Fitness improves your chances of being selected.
- Crossover: When two selected models exchange genetic information, their kids receive traits from both parents. It replicates genetic material recombination in biological reproduction.
- Mutation: Random modifications are introduced into the offspring’s genetic code to increase diversity and explore new parts of the solution space.
- Replacement: The following generation comprises the children and some surviving parent models. The method refines the population across subsequent generations, eventually convergent on better-fitting models.
- Polynomials, logarithmic, and trigonometric functions are mathematical expressions found within chromosomes. The method seeks the function and parameter combination that best captures the underlying relationships in the data.

MGGP is especially useful when the interactions between variables are complex and cannot be effectively captured by traditional linear models. Its ability to evolve complex mathematical expressions makes it useful for symbolic regression problems, system identification, and comprehending complex phenomena in various domains [33–35].

#### 2.6.2 XGBoost.

Extreme Gradient Boosting, or XGBoost, is a well-known and advanced machine learning method that is known for being very good at making predictions. XGBoost is a renowned ensemble learning descendant that employs an enhanced boosting strategy that optimizes the performance of decision tree-based models. To do this, a sequence of weak learners, often represented as decision trees, are created one after another and then combined to form a powerful and precise forecasting model [36]. It is a powerful approach since it can effortlessly find and use complex associations within datasets. It is an excellent option for dealing with massive, complex data structures. The system can make predictions, and its feature importance analysis assists users in identifying and ranking the most pertinent characteristics in their datasets. XGBoost also features regularization algorithms, which prevent the model from overfitting and improve its capacity to adapt to new data conditions [37,38].

Since it performs well and can be employed in many circumstances, it is becoming popular for many predictive modeling tasks. Its wide use is because it consistently performs better, whether it is in regression or classification jobs. XGBoost is still an essential algorithm in predictive analytics, even though people who work with machine learning are looking for more durable solutions that can handle the complexities of the real world.

## 3.0 Results and discussion

###  3.1 Nanofluid properties

**Fig 3:**
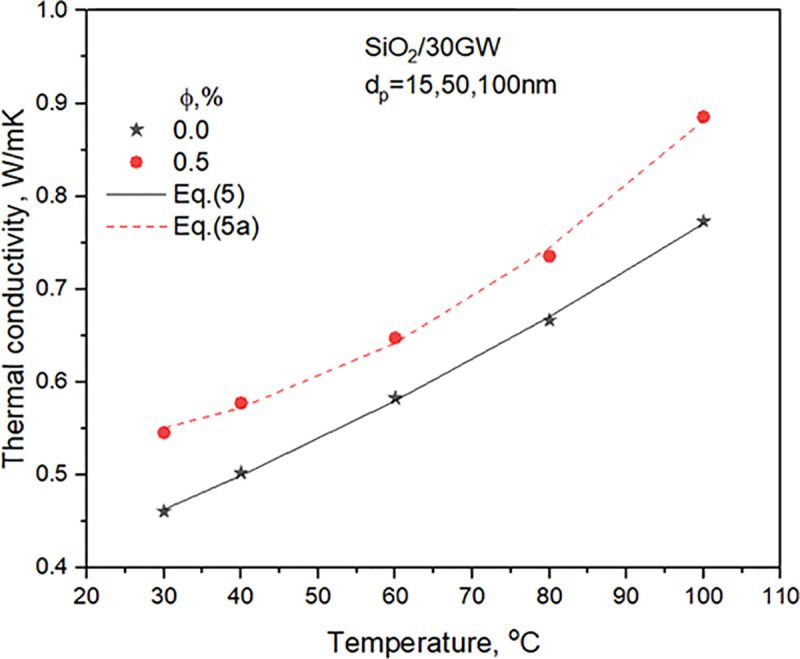
Variation of 30GW and SiO_2_ nanofluid TC with temperature.

**Fig 4:**
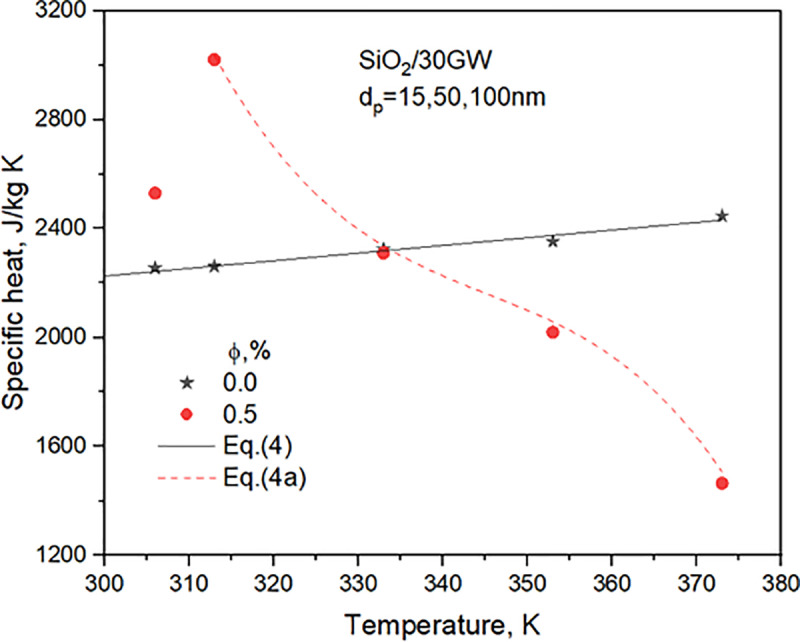
Variation of 30GW and SiO_2_ nanofluid specific heat with temperature.

**Fig 5:**
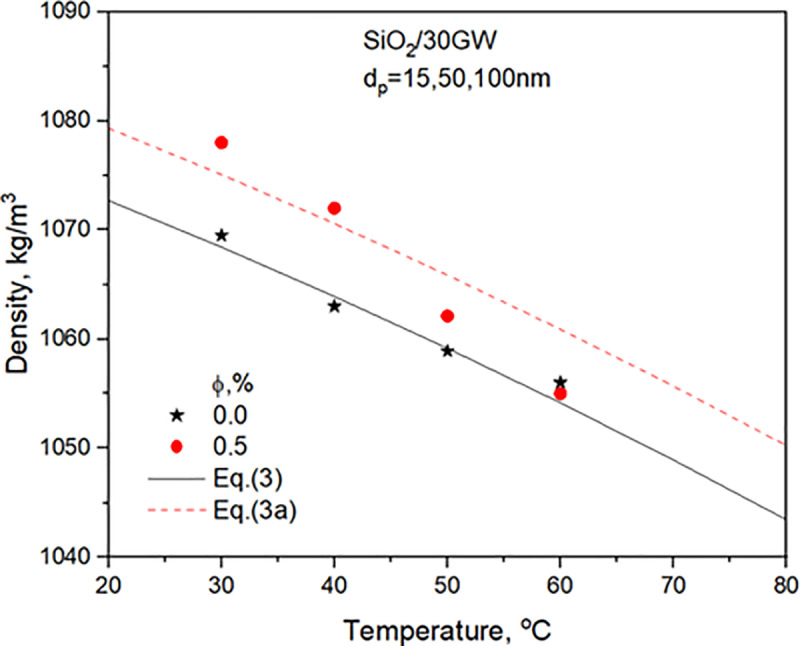
Variation of 30GW and SiO_2_ nanofluid and density with temperature.

**Fig 6:**
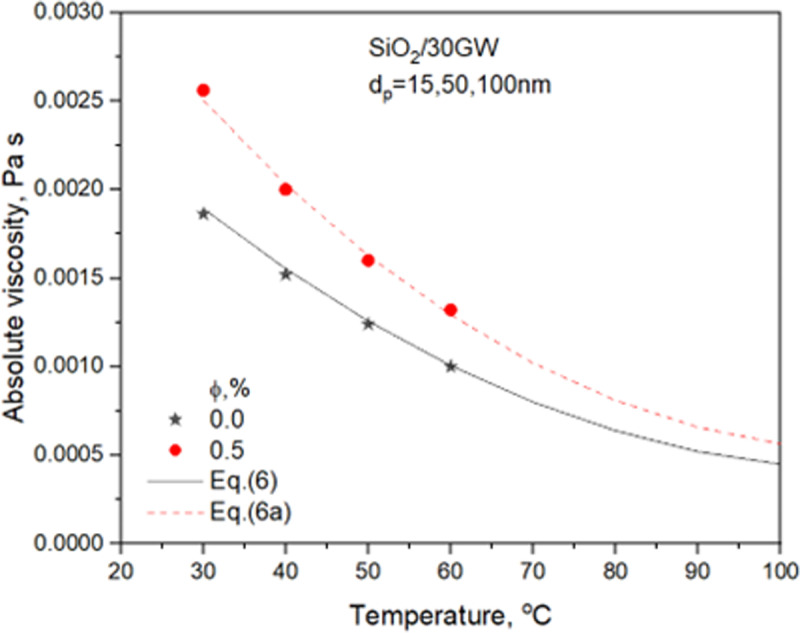
Variation of 30GW and SiO_2_ nanofluid viscosity with temperature.


The thermophysical properties of 30GW and SiO_2_ nanofluids for two concentrations are detailed in **Sharma et al.** [29]. The property relations used in the heat transfer analysis are presented. Figs 3–6 compares properties, i.e., TC, SH, density, and viscosity of 0.5% concentration nanofluid with the values of the base liquid. The nanofluid’s TC, density, and viscosity are more significant than the base liquid. However, the nanofluid SH shows a considerable variation with temperature. It shows a greater value than the base liquid for temperatures lower than 40^o^C and decreases to lower than the base liquid values after that.

$${\;\;\rho \;\;}_{\text{bf}}\text{=1080}.5081-\text{(0}.366636)({\text{T}}_{\text{b}}\text{)}-\text{(0}.00121)({\text{T}}_{\text{b}}{\text{)}}^{\text{2}}$$ (3)

$$\begin{array}{c} {\;\;\rho \;\;}_{\text{nf}}\text{=1087}.1-\text{(0}.3648)({\text{T}}_{\text{b}}\text{)}-\text{(0}.0012)({\text{T}}_{\text{b}}{\text{)}}^{\text{2}}\;\text{holds in the range of }\varphi \text{ = 0}.5\;\;\\ \;\;\text{ in the range 20}\circ \text{C }\leq T\leq \text{ 100}\circ \text{C}. \end{array}$$ (3a)

$$\begin{array}{c} \begin{array}{c} {\text{C}}_{\text{p,bf}}=\text{2388}.77633-\text{ 467}.98957 exp (-{\text{T}}_{\text{b}}\text{/ 22}.7548) valid in the range 30\circ \text{C}\leq T\leq \text{100}\circ \text{C}\\ \text{with a maximum deviation of 1}.52\%\text{.} \end{array} \end{array}$$ (4)

$$\begin{array}{c} \begin{array}{c} {\text{C}}_{\text{p,nf}}\text{=}-\text{591634}.9-\text{5096}.03({\text{T}}_{\text{b}}+\text{273)}+\text{14}.7212({\text{T}}_{\text{b}}\text{+273}{\text{)}}^{\text{2}}-\text{0}.01421({\text{T}}_{\text{b}}+\text{273}{\text{)}}^{\text{3}}\text{ holds}\;\text{in}\;\text{range}\;\text{of}\;\\ \varphi =\text{0}.5\backslash \%\;\;\;\;\text{ , 40}<{\text{T}}_{\text{b}}<\text{100}\circ \text{C well}\;\text{within}\;\text{peak}\;\text{deviation}\;\text{of}\;<\text{5\textbackslash{}\%}. \end{array} \end{array}$$ (4a)

$${\text{k}}_{\text{bf}}=\text{0}.36803+\text{0}.00279({\text{T}}_{\text{b}}\text{)}+\text{1}.23689E-5(T\text{b}{\text{)}}^{\text{2}}$$ (5)

$${\text{k}}_{\text{nf}}=\text{0}.5343-\text{(7}.281E-\text{4)(}{\text{T}}_{\text{b}}\text{)}+\text{(4}.20224E-\text{5)(}{\text{T}}_{\text{b}}{\text{)}}^{\text{2}}\text{, valid}\;\text{for}\;\varphi =\text{0}.5\;\;\;\;\;\text{ , Max}\;\text{dev}<\text{1}.3\;\text{.}$$ (5a)

$${\;\;\mu \;\;}_{\text{bf}}=\text{0}.00318-\text{4}.9625e-\text{5*}{\text{T}}_{\text{b}}\text{+2}.23214e-\text{7*}{{\text{T}}_{\text{b}}}^{\text{2}}$$ (6)

$$\begin{array}{c} \begin{array}{c} {\;\;\mu \;\;}_{\text{nf}}=\;\;\mu \;\;{\text{b}}_{\text{f}}\text{*0}.9794*({\text{T}}_{\text{b}}\text{/80}{\text{)}}^{\text{(}-\text{0}.0436)}\;{\text{(1}+\varphi \text{/100)}}^{\text{(53}.21)}*\text{(1}+\text{dp/80)}\;\text{(0}.01519)\;\\ \text{valid for 0}.5\leq \varphi \leq \text{1}.0\%\;\;\;\text{ in the temperature range 30 }\;\;\circ \;\;\text{ C}\leq {\text{T}}_{\text{b}}\leq \text{60 }\;\;\circ \;\;\text{ C}. \end{array} \end{array}$$ (6a)

### 3.2 Property enhancement ratio

**Fig 7:**
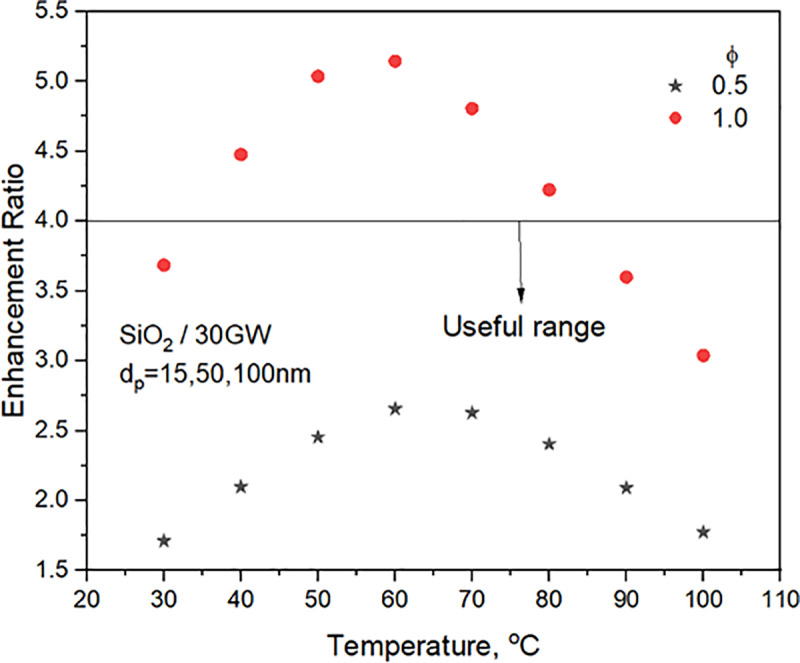
Variation of viscosity to TC Enhancement Ratio, Eq. (7) with temperature.


The Property enhancement ratio (ER) is calculated by comparing the increase in viscosity to the rise in TC relative to the base liquid and given by **Eq. (7). Prasher et al.** [39] undertook an order of magnitude analysis to evaluate the relative advantage of nanofluids over the base liquid heat transfer. The condition arrives with the properties for fully developed flow in a tube centering over the nanofluid pressure drop. Later, **Garg et al.** [40] used their experimental data on TC and viscosity to arrive at a similar condition with a slight change in the value. Accordingly, the value of ER for favorable nanofluid heat transfer benefit should be lower than 4.0 and 5.0, respectively, according to **Prasher et al.** [39] and **Garg et al.** [40] in the operating range of temperatures. The variation of ER for the nanofluid concentrations of 0.5 and 1.0% with temperature is shown in **Fig 7**.

$$Enhancementratio,ER=\left[\left(\mu_{nf}-\mu_{bf}\right)k_{bf}\right]/\left[\left(k_{nf}-k_{bf}\right)\mu_{bf}\right]$$ (7)

### 3.3 Mouromtseff number

**Fig 8:**
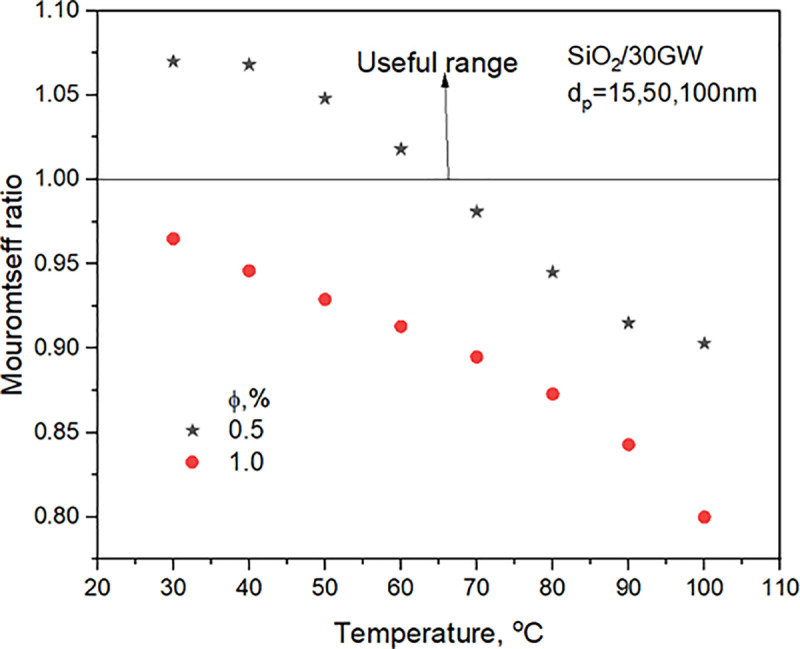
Comparison of SiO_2_ nanofluid Mo ratio for two concentrations with temperature.

The Mouromtseff number predicts heat transfer enhancement of nanofluid evaluated with the fluid properties. **Fig 8** shows the heat transfer capability of the SiO_2_ nanofluid compared to base fluid for turbulent flow in a tube. As can be seen, the ratio of the M_O_ number is more significant than one in a specific temperature range for the concentration envisaged, which shows the advantage of utilizing SiO_2_ nanofluid for heat transfer applications. The M_O_ number has a positive correlation with temperature and a negative correlation with concentration. The decrease may be attributed to the non-significant TC and density increase compared to base fluid. The maximum MO number ratio is estimated to be 1.08 at 60^o^C for 0.5 vol. %.

### 3.4 Heat transfer characteristics

#### 3.4.1 Heat transfer coefficient.

**Fig 7** predicts heat transfer enhancements with SiO_2_/30GW nanofluid in a wide range of temperatures for 0.5% concentration. For 1.0% concentration, enhancements in turbulent heat transfer coefficients are predicted for nanofluid temperatures <40^o^C or> 80^o^C. The experimental operating range lies between 40 and 60^o^C. **Fig 8** supports the observations in **Fig 7** that SiO_2_/30GW of 0.5% concentration predicts heat transfer enhancements. However, from **Fig 8**, it can be observed that heat transfer enhancements are possible with 0.5% concentration nanofluid for temperatures <65^o^C. Hence, experiments are undertaken only with 0.5% SiO_2_/30GW nanofluid concentration with operating temperatures <65^o^C. Hence, experiments with nanofluid are undertaken in the valuable range observed.

#### 3.4.2 Convective heat transfer.

**Fig 9:**
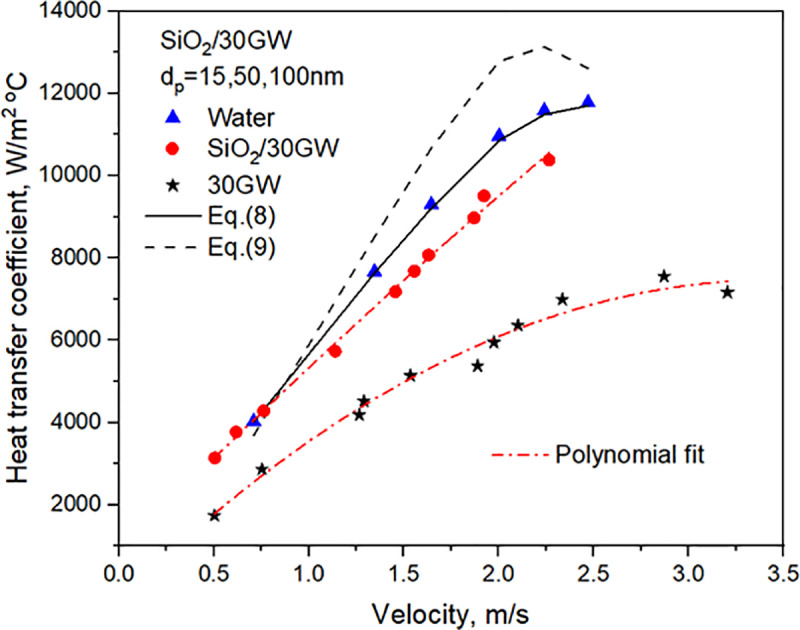
Comparison of turbulent water data with standard relations in the literature.


Before conducting systematic heat transfer experiments with SiO_2_ nanoﬂuid, the experimental setup was tested with water and base liquid 30GW. The results obtained with water serve as a basis for comparison with other liquids, as shown in **Fig 9**. For the range of flow velocities in the tube, the relations of **Dittus-Boelter** [41] and **Gnielinski** [42], respectively given by **Eqs. (8) and (9)** are applicable for estimating the HTC of Water due to their range of applicability.

$$\begin{array}{c} Nu=0.023{Re}^{0.8}{\mathrm{Pr}}^{0.4}\text{valid\textbackslash{} for}0.6\leq Pr\leq 160;Re\geq 10000;(L/D)>60 \end{array}$$ (8)

$$Nu=\left[\frac{\left(\frac{f}{2}\right)(Re-1000)\mathrm{Pr}}{1.07+12.7{\left(\frac{f}{2}\right)}^{0.5}\left({\mathrm{Pr}}^{\frac{2}{3}}-1\right)}\right]\left[1+{\left(\frac{D_{h}}{L}\right)}^{2/3}\right]{\left[\frac{\mu }{\mu_{s}}\right]}^{0.11}$$ (9)

where Darcy friction factor $f={\left[1.58ln(Re)-3.82\right]}^{-2}$ hold only in the range of 2300 < Re < 10^6^, 0.5 < *Pr* < 2000.

The experimental data of water in the 8800–36000 range of Reynolds number are observed to be in close agreement with the values estimated with **Eq. (8)** of **Dittus-Boelter** [41]. The experimental data of Nusselt for Water lies in the transition-turbulent range of Reynolds, i.e., 8800–36000. **Eq. (9)** considers the effect of entry length reflected by the term $\left[1+{\left(\frac{D_{h}}{L}\right)}^{2/3}\right].$ The influence of the term $\left[1+{\left(\frac{D_{h}}{L}\right)}^{2/3}\right]$ on Nusselt number enhancement in the turbulent range of Reynolds number is about 4%, and that of the viscosity ratio, ${\left[\frac{\mu }{\mu_{s}}\right]}^{0.11}$, is about 2%.

The water data underestimated the values with the **Eq. (9)** of **Gnielinski** [42] by a maximum of 11.94% at the higher Reynolds number, as can be observed from **Fig 9**. Possibly, the flow of water in the flexible tube of 1.0m length before its entry into the test section enabled attainment of turbulence before it entered the test section.

The heat transfer data of aqueous glycerol, 30GW, lie in the 3900–19000 range of Re. The experimental values of Nu are compared with Eq. (8), which is valid for pure liquids and Re greater than 10,000. The experimental values of 30GW deviate from Eq. (8) by a maximum of 15.06% for lower Reynolds numbers.

**James** [43] has undertaken heat transfer experiments for flow in various geometries with aqueous glycerol of 48.5 and 65.7% concentration by weight. The author has developed a relation for the estimation of Nu for the flow of aqueous glycerol in a tube as


$$Nu=\frac{0.104{Re}^{0.0016Pr+0.75}}{\left(2.05+1.62\exp^{-1}\right)}\frac{{\mathrm{Pr}}^{0.4}}{\exp^{0.0134Pr}}{\left(\frac{\mu_{B}}{\mu_{w}}\right)}^{213{Re}^{-0.9}}$$ (10)

The heat transfer coefficients estimated with **Eq. (10)** predict the present data of 30% vol. concentration (25.4 vol/vol%) glycerol closely, as shown in **Fig 9**. It can be observed from **Fig 9**. that the heat transfer coefficients with SiO_2_/30GW nanofluid is lower compared to values with pure water, though not significant. The nanofluid is relatively stable for long durations of time giving scope for practical applications especially in heat exchangers where higher temperatures are encountered, since water has a lower boiling point.

**Fig 10:**
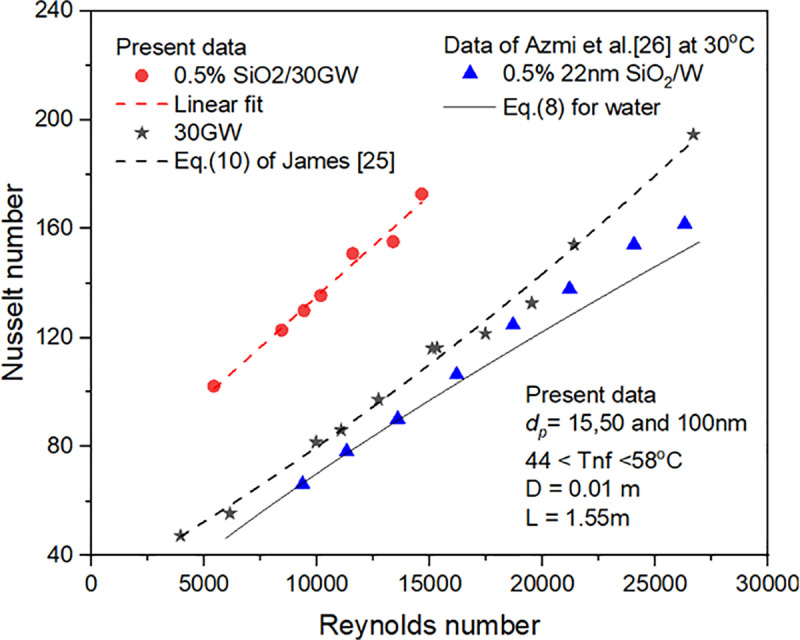
Comparison of 0.5% concentration SiO_2_ nanofluid dispersed in water and 30GW.

The experimental values of the Nusselt number are shown in Fig 10 for SiO_2_ nanofluid in base liquids 30GW and water. **Azmi et al.** [44] conducted SiO_2_/water nanofluid experiments for 0.5% concentration at 30^o^C. The present data of 0.5% concentration SiO_2_/30GW nanofluid is shown compared with the data of **Azmi et al.** [44] in Fig 10. The Nusselt numbers of SiO_2_/30GW nanofluid are observed to be greater than the values of the base liquid and SiO_2_/W nanofluid. The lower SiO_2_/30GW nanofluid TC values might be the reason for the higher Nusselt numbers obtained. The experimental Nusselt numbers of SiO_2_ nanofluid in base liquid water are more significant than the experimental values obtained.

### 3.5. Pressure drop

**Fig 11:**
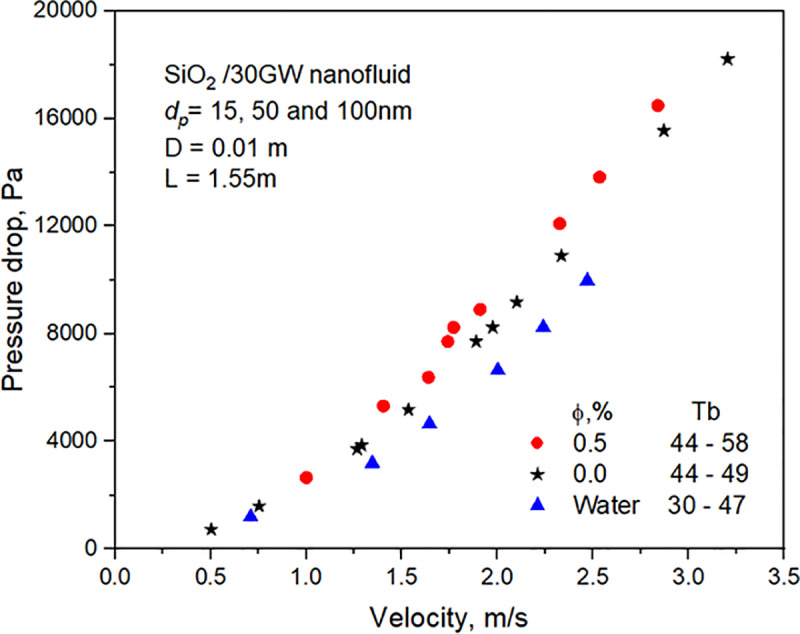
Variation of pressure drop across the test section.

**Fig 12:**
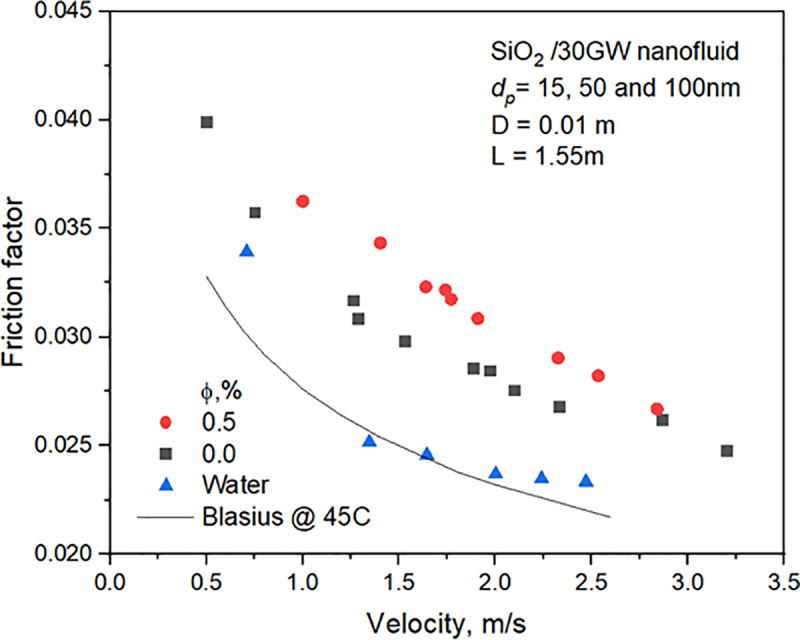
Variation of friction factor of the three fluids at different velocities.

The pressure drop of nanoﬂuid ﬂowing in the copper tube of 1.55m length is measured under different flow conditions. Fig 11 depicts the experimental data of water pressure drop, 30GW, and SiO_2_/30GW nanofluid. The pressure drops increase with liquid velocity and concentration. The friction factor of water obtained from experiments is compared with the Blasius equation with properties estimated at 45^o^C. The friction factor of 30GW and SiO_2_ nanofluid estimated with the Darcy equation decreases with an increase in liquid velocity, as shown in **Fig 12**, and increases with concentration.

### 3.6 ML-based model prediction

The data acquired through lab-based tests was employed to develop prognostic models for friction factor and heat transfer. The first step is to collect data from tests that were done in a lab. This data set usually details many things, like the qualities of the fluid, its flow conditions, temperature, and pressure drop. This data is used as a base for training and testing the predictive models. A lot of the time, raw experimental data needs to be preprocessed to get rid of missing numbers, outliers, and other problems. Python tools like Pandas are used to clean and organize the dataset to prepare it for model training. EDA is essential for knowing how the dataset is put together. Python libraries like Matplotlib and Seaborn make it easy to visualize how different factors are related, spot trends and learn more about the patterns beneath the data.

**Fig 13:**
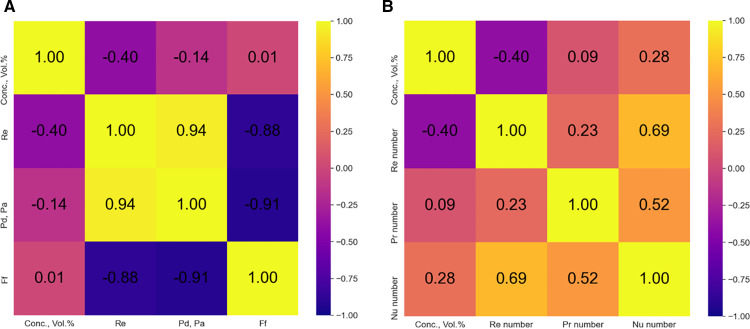
Correlation heatmap for (a) Friction factor (b) Nu number.

The correlation heatmaps developed for the data employed in the present study are depicted in **Fig 13a** for friction factor and **Fig 13b** for Nu number. It can be observed that in the case of pressure drop, a good correlation of 0.94 exists between Re and pressure drop, indicating that higher Re will result in higher pressure drop. Conversely, a highly negative correlation value of -0.88 was observed between Re number and friction factor and -0.91 between pressure drop and friction factor. On the other hand, a positive correlation of 0.69 was observed between Re and Nu, as depicted in **Fig 13b**.

The descriptive statistical analysis of the data is listed in Table 3. The descriptive analysis offers an in-depth look at the nanofluid-based heat transfer data, considering the concentration, Prandtl number, Reynolds number, Nusselt number, pressure drop, and friction factor. The mean Nu model has been determined to be 105.17 with a standard deviation of 37.11, indicating substantial variability around the mean. With a kurtosis of -0.91, the data is spread relatively flat, with more petite tails than a normal distribution. When kurtosis is negative, the data is less skewed and has fewer significant values than a normal distribution. If the skewness is 0, on the other hand, it means that the distribution is almost symmetric, meaning that the tails on both sides are about the same length [45,46].

The mean for the friction factor model is 0.031, with a standard deviation of 0.004, indicating a restricted range of values. Compared to a normal distribution, the kurtosis of -0.557 indicates a distribution with heavier tails and a flatter peak. The skewness of 0.761 suggests a slight rightward skew, suggesting a somewhat prolonged right-side tail. The kurtosis and skewness values in this context provide insights into the properties of the Nu and friction factor distributions, assisting investigators in making informed decisions and interpretations of the present work. Table 1 presents the descriptive statistical analysis.

**Table 1:** Descriptive statistical analysis.

Stat.	Nu model				Friction factor model			
	Conc., Vol.%	Re	Pr	Nu	Conc., Vol.%	Re	Pressure drop	Friction factor
Count	19	19	19	19	19	19	19	19
Mean	0.26	10462.99	4.02	105.17	0.263	10462.99	5214.04	0.031
Std deviation	0.256	4708.48	0.71	37.11	0.256	4708.48	3273.25	0.004
Min	0	3964.09	2.92	47.34	0	3964.09	664.43	0.027
25%	0	5821.20	3.62	75.57	0	5821.20	2458.40	0.028
50%	0.5	10174.31	3.98	105.01	0.5	10174.31	5182.56	0.030
75%	0.5	14015.63	4.32	131.29	0.5	14015.63	7640.96	0.034
Max	0.5	19531.17	5.65	172.76	0.5	19531.17	10896.67	0.039
Kurtosis	-2.235	-0.93	0.28	-0.95	-2.235	-0.93	-1.09	-0.557
Skewness	-0.115	0.21	0.59	0.00	-0.115	0.21	0.14	0.761

**Fig 14:**
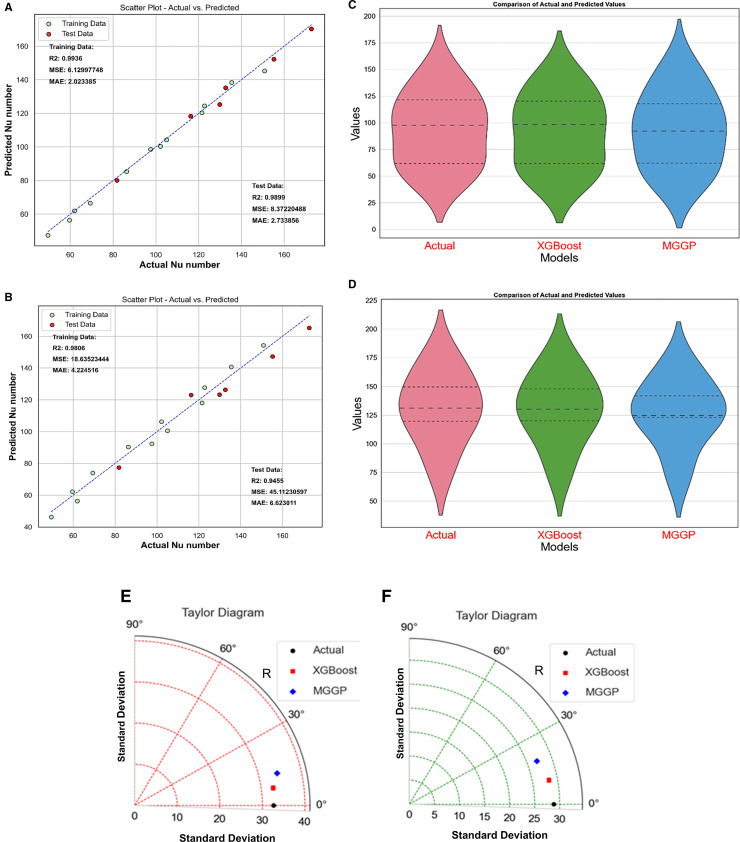
Nu number model. (a) XGBoost predicted vs. actual (b) MGGP predicted vs. actual. (c) violin plots for the training phase, (d) violin plots for the test phase. (e) Taylor diagram for the training phase (f) Taylor diagram for the test.

The Nu and friction factor prognostic model was developed after the data analysis with correlation heatmap and descriptive statistical analysis. The data was split into a 70:30 ratio for model training and random testing. Once the models were developed, these were used for prediction. **Fig 14** shows the results of the Nu models. **Fig 14a** compares actual and model predicted values for the XGBoost model, while **Fig 14b** illustrates the comparative analysis of MGGP based Nu model. It can be observed that both modes fare well, but the XGBoost-based model is more robust. The models were also compared using violin plots. Violin plots are depicted in **Fig 14c** for the model training phase and **Fig 14d** for the model test phase. These violin plots compare the distribution, central tendency, and variance of predicted values for XGBoost and MGGP machine learning models. The breadth and form of each “violin” reflect differences in model precision, consistency, and robustness. Outliers and statistical metrics improve insights, allowing researchers to evaluate and choose the best model for their study. Violin plots provide an easy-to-understand and brief way of assessing and comparing the performance of various ML approaches [47]. Both during mode training and the model testing phase, the violin plot of the based model almost mimics the actual data, indicating the superior performance of XGBoost-based models.

The performance of prognostic models was also compared using Taylor’s diagram. Taylor diagrams provide a valuable depiction of how much each model matches observed data by clearly displaying correlation, root mean square error (RMSE), and standard deviation. The concentric circles and radial lines make judging model correctness and spread easy. Taylor diagrams provide researchers with a succinct and intuitive manner to identify the prediction strengths and shortcomings of XGBoost and MGGP [48,49]. **Fig 14e** depicts Taylor’s diagram for the model training phase, while **Fig 14f** illustrates model comparison during the model Test phase. Hereto, it was observed that XGBoost-based models were more accurate compared to MGGP-based models, as they were close to the actual point over the diagrams in both cases.

The statistical values for each model are marked on the comparative plots, i.e., **Figs 14a** and **14b**. During the model test, the coefficient of determination (R^2^) value of the XGBoost-based model was 0.9899, but it was reduced in the case of the MGGP-based model to 0.9455. However, the difference was only negligible. The mean squared value was 8.37222 for XGBoost, while it increased in the MGGP model to 45.122. Similarly, the mean absolute error (MAE) value was higher (6.623) in the case of MGGP than in XGBoost at 2.733. The statistical analysis also reveals that XGBoost performed superior to MGGP in predicting Nu.

**Fig 15:**
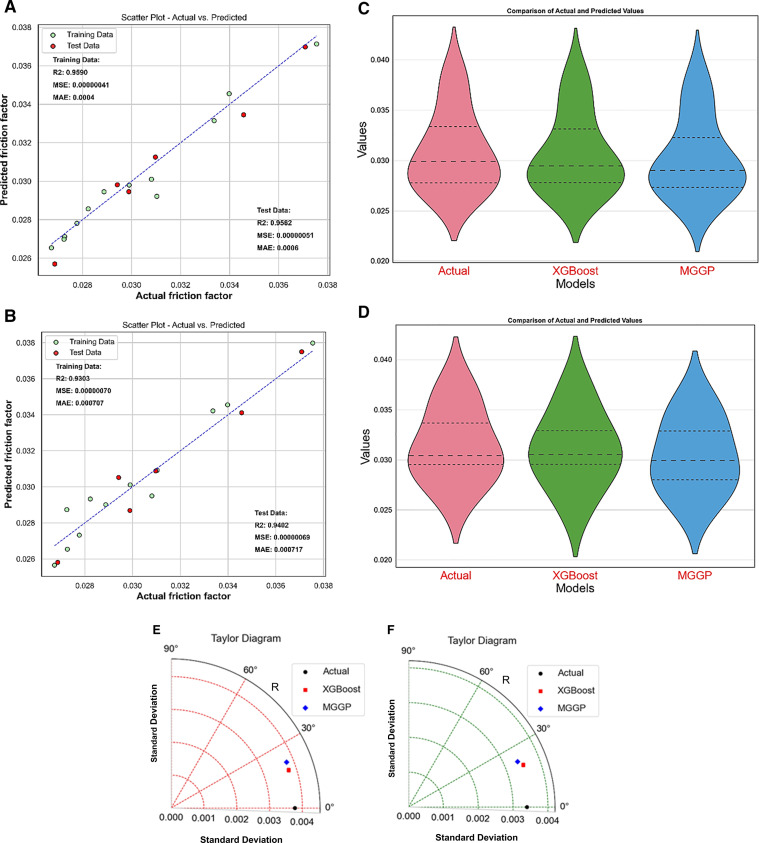
Friction factor model. (a) XGBoost predicted vs. actual (b) MGGP predicted vs. actual (c) violin plots for the training phase, (d) violin plots for the test phase (e) Taylor diagram for the training phase (f) Taylor diagram for the test phase.

The predictive model for friction factor was established after data analysis with correlation heatmap and descriptive statistical analysis. The data was randomly divided into a 70:30 ratio for model training and testing. Once the model was created, it was employed to make predictions. **Fig 15** depicts the friction factor model results. **Fig 15a** shows a comparison of actual and predicted values for the XGBoost model, whereas **Fig 15b** shows a comparison of the MGGP-based friction factor model. Both models perform admirably, but the XGBoost-based model is more resilient. Violin plots were also used to compare the models. **Fig 15c** shows violin plots for the model training phase, and **Fig 15d** shows violin plots for the model test phase. The distribution, central tendency, and variance of predicted values for the XGBoost and MGGP ML models are compared in these violin charts. The violin plot of the XGBoost-based model almost replicates the actual data during both the mode training and model testing phases, suggesting the improved performance of XGBoost-based models.

Taylor’s diagrams were also used to compare the performance of prognostic models. Taylor diagrams, which represent correlation, RMSE, and standard deviation, give a valuable visual depiction of the amount to which each model aligns with observed data [50]. The concentric circles and radial lines make judging model correctness and spread simple. Taylor diagrams allow researchers to quickly and easily identify the prediction strengths and weaknesses of XGBoost and MGGP. **Fig 15e** exhibits Taylor’s diagram during soithe model training phase, whereas **Fig 15f** depicts model comparison during the model testing phase. XGBoost-based models were more accurate than MGGP-based models since they were close to the actual point over the diagrams in both cases.

In the comparative plots, **Figs 15a** and **15b**, the statistical values for each model are indicated. The XGBoost-based model’s coefficient of determination (R^2^) value was 0.9582 during model training; however, it was low in the case of the MGGP-based model at 0.9402 during the model test phase. However, the difference was insignificant. The XGBoost model had a mean squared value of 0.0000051, whereas the MGGP model had a value of 0.0000069. Similarly, the mean absolute error (MAE) for MGGP was more significant (0.0006) than for XGBoost, which was 0.000717, during model testing. The statistical research also showed that XGBoost outperformed MGGP in predicting Nu.

## 4. Conclusions

The heat transfer and flow behavior of aqueous SiO_2_/30GW nanofluids flowing through a straight horizontal pipe under turbulent Reynolds number conditions has been investigated experimentally. The influence of SiO_2_ nanoparticles equal dispersion of 15, 50, and 100nm size in aqueous glycerol of 30% concentration by weight on properties and their combined effect on the heat transfer coefficients are investigated. The heat transfer coefficient and friction factor data were analyzed based on experiment thermophysical properties correlations. It was employed for prognostic model development using XGBoost and MGGP to model and predict the complex and nonlinear data acquired during experiments. Results indicate a steady heat transfer increase with volume fraction. EG-based nanofluids exhibited moderately higher heat transfer coefficients than Glycerol-based NFs. The following are the conclusions:

- The density, TC, and viscosity of nanofluid show higher values than those of base liquid. The specific heat shows higher values for temperatures lower than 330K and decreases rapidly afterward.
- The values of the property enhancement ratio and Mouromtseff numbers indicate heat transfer enhancements with a 0.5% nanofluid concentration and operating temperatures lower than 60^o^C. The convective heat transfer coeﬃcients are observed to show a descending order of magnitude with water, base liquid 30GW, and SiO_2_/30GW nanofluid, respectively. However, the Nusselt numbers show an opposite trend due to lower TC values than water.
- The pressure drop of the three liquids, water, 30GW, and nanofluid, increases with the flow velocity. Pressure drops increase with liquids having higher viscosity. The friction factor estimated using the Blasius equation for water at an average temperature of 45^o^C agrees with the experimental data. The friction factors are observed to increase with the liquid’s viscosity.
- During the model test, the coefficient of determination value of the XGBoost-based model was 0.9899, but it was reduced in the case of the MGGP-based model to 0.9455. However, the difference was only negligible. The mean squared value was 8.37222 for XGBoost, while it increased in the MGGP model to 45.122. Similarly, the mean absolute error (MAE) value was higher (6.623) in the case of MGGP than in XGBoost at 2.733. The statistical evaluation, Taylor diagrams, and violin plots helped determine that XGBoost was superior to MGGP in the present work.
- These findings have practical significance for heat exchanger design and cooling systems. The improved heat transfer with SiO_2_ nanofluids can boost system efficiency, reduce energy use, and allow for smaller, more effective equipment. The friction factor data also helps optimize flow rates, improving heat transfer without increasing pressure drop. This makes nanofluids valuable for industries requiring efficient cooling, like automotive, HVAC, and electronics.

### Potential limitations of the study

The study employs a fixed 7:3 water-glycerol mixture as the base fluid, which may restrict the findings’ applicability to other fluids with differing heat transfer and flow characteristics. It focuses on SiO2 nanoparticles at sizes of 15, 50, and 100 nm with a concentration of 0.5%, potentially limiting the insights applicable to varying particle sizes or concentrations. Additionally, the research targets the transition-turbulent flow regime, neglecting laminar conditions and thereby providing an incomplete picture of nanofluid behaviour across all flow types. The machine learning models developed were trained on data specific to the experimental conditions, which may hinder their predictive capability in different settings. Furthermore, the study only evaluated XGBoost and MGGP models, indicating the need to investigate other machine learning approaches that could yield improved predictions and broader applicability.

### Future research directions

Future research should explore a variety of nanoparticles, such as Al_2_O_3_ and TiO_2_, and utilize a broader range of concentrations to enhance the understanding of heat transfer and flow dynamics. It is essential to test different base fluids or mixtures that are relevant to industrial applications to evaluate their interactions with nanoparticles and potential benefits. Additionally, expanding the study to include all flow regimes—laminar through fully turbulent—would provide a more thorough analysis of nanofluid performance. Employing advanced hybrid machine learning techniques that combine deep learning with ensemble models could improve predictions of complex, nonlinear datasets. Conducting sensitivity and uncertainty analyses would further clarify model reliability and the influence of varying experimental conditions. Lastly, validating these models in real-world systems, such as heat exchangers, will be crucial for confirming their practical effectiveness in industrial settings.

ML techniques significantly enhance predictive modeling for nanofluid data. Deep Neural Networks are effective at identifying complex, nonlinear relationships, while Long Short-Term Memory networks excel in making predictions based on time-dependent data. Support Vector Machines improve model generalization, particularly with smaller datasets. Bayesian Optimization optimizes model hyperparameters, increasing accuracy, and AutoML streamlines model selection and optimization, yielding optimal results with minimal manual effort. Collectively, these methods greatly enhance prediction capabilities in intricate systems.

## Nomenclature

**Abbreviations**

**Table:** 

D d_p_	Tube diameter, mm Particle diameter, nm
DLS	Dynamic light scattering
EDX	Energy dispersive X-ray spectroscopy
EG	Ethylene glycol
FESEM	Field emission scanning electron microscopy
FTIR	Fourier transform infrared spectroscopy
f	Friction factor
h	Heat transfer coefficient, W/m^2-^K
*k*_f_	Thermal conductivity test ﬂuid.
L	Length of the tube, mm
MAE	Mean absolute error
MGGP	Multi-gene genetic programming
ML	Machine learning
Mo	Mouromtseff number
Nu	Nusselt number
ER	Property enhancement ratio
Pr	Prandtl number
R^2^	Coefficient of determination
Re RMSE	Reynolds number Root mean square error
SH	Specific heat, J/kg K
SiO_2_	Silicon dioxide
T_b_	Base fluid temperature, °C
TC	Thermal conductivity, W/m-K
TPS	Transient Plane Source
UV	Ultraviolet-visible Spectroscopy
XGBoost	Ensemble-based extreme gradient boosting

**Greek Symbols**

**Table:** 

ρ	Density, kg/m^3^
	Volume concentration, %
µ	Dynamic viscosity, mPa.s

**Subscripts**

**Table:** 

SiO_2_	Silicon oxide
GW	Glycerol water
f	Fluid
nf	Nanofluid
bf	Base fluid
